# Microscopic messengers: Extracellular vesicles shaping gastrointestinal health and disease

**DOI:** 10.14814/phy2.70292

**Published:** 2025-04-01

**Authors:** Zhantao Yu, Kevin A. Swift, Madeline A. Hedges, Arianne L. Theiss, Sarah F. Andres

**Affiliations:** ^1^ Division of Gastroenterology and Hepatology, Department of Medicine and the Mucosal Inflammation Program University of Colorado School of Medicine Aurora Colorado USA; ^2^ Department of Pediatrics, Pediatric GI Division, School of Medicine Oregon Health and Science University Portland Oregon USA; ^3^ Department of Neonatology, School of Medicine Oregon Health and Science University Portland Oregon USA; ^4^ Rocky Mountain Regional Veterans Affairs Medical Center Aurora Colorado USA

**Keywords:** bacteria, exosome, inflammation, intestine

## Abstract

The field of extracellular vesicles (EVs) is advancing rapidly, and this review aims to synthesize the latest research connected to EVs and the gastrointestinal tract. We will address new and emerging roles for EVs derived from internal sources such as the pancreas and immune system and how these miniature messengers alter organismal health or the inflammatory response within the GI tract. We will examine what is known about external EVs from dietary and bacterial sources and the immense anti‐inflammatory, immune‐modulatory, and proliferative potential within these nano‐sized information carriers. EV interactions with the intestinal and colonic epithelium and associated immune cells at homeostatic and disease states, such as necrotizing enterocolitis (NEC) and inflammatory bowel disease (IBD) will also be covered. We will discuss how EVs are being leveraged as therapeutics or for drug delivery and conclude with a series of unanswered questions in the field.

## Graphical abstract

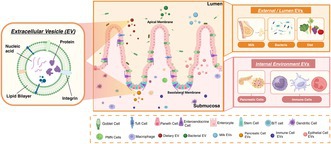

## 1 INTRODUCTION

### 1.1 Extracellular vesicles

Extracellular vesicles (EVs) are double‐lipid membrane enclosed, nano‐sized carriers of biological information, including proteins, nucleic acids, lipids, and macromolecules (Welsh et al., 2024). These acellular structures are produced by most cells, including mammalian (Anderson, 1969; Mathieu et al., 2019; Wolf, 1967), bacterial (Briaud & Carroll, 2020; Brown et al., 2015; Chatterjee et al., 1959; De, 1959; Sartorio et al., 2021), and plant cells (Jensen, 1965; Lian et al., 2022). EVs are produced and released through various cellular processes, which influence their cargo and cellular destination (Welsh et al., 2024). For the sake of simplicity, we will refer to any membrane‐enclosed vesicle as an EV throughout this review, including exosomes, outer membrane vesicles (OMV) from bacteria, and plant‐derived vesicles from various sources.

Interest in EVs as biological messengers, modulators of health and disease, biomarkers of pathology, and even as drug delivery vehicles has surged in recent years (Bazzan et al., 2021). To establish consistency in terminology and experimental methodology, the minimal experimental requirements for studies of EVs were first established in 2014 to provide guidelines for EV research and to strengthen the rigor in the field (Lotvall et al., 2014). These guidelines delineate methods of EV isolation and characterization, as well as terminology for defining EV structures and functions. The characterization methods include electron microscopy paradigms to examine vesicle morphology, quantification methods for particle size and concentration, methods of measuring particle cargo, including protein types and concentration, nucleic acids, and lipid content (Welsh et al., 2024). Although there is no single or small set of markers that define all EVs, authors must report their findings with as much detail as possible to allow for transparency in data collection and comparison with other studies. In this review, we endeavor to include information about the EV types used in the included studies. We also point our readers to the MISEV guidelines (Lotvall et al., 2014; Thery et al., 2018; Welsh et al., 2024) for more information about EV methodology and data reporting.

### 1.2 The lower gastrointestinal tract

**FIGURE 1:**
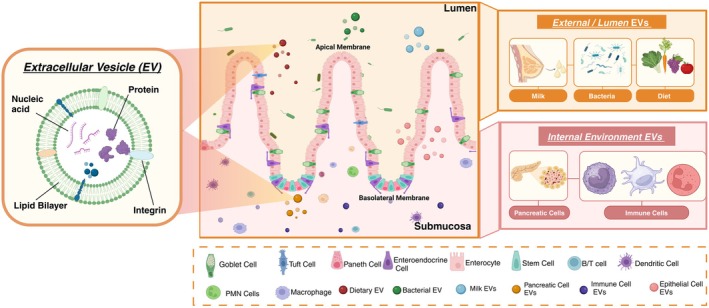
The intestinal epithelium interacts with extracellular vesicles (EVs) from internal and external sources. EVs are double‐lipid membrane‐enclosed nanoparticles that carry biological information and are produced by a variety of cell types and sources. Given the barrier nature of the intestinal epithelium, it interacts with EVs from numerous internal and external sources, influencing health and disease states.

The lower gastrointestinal tract (small intestine and colon) is responsible for myriad functions, including serving as a barrier between the inside and the outside world, nutrient digestion and absorption, water absorption, hormone secretion, and immune functions (Chelakkot, Ghim, & Ryu, 2018; Soderholm & Pedicord, 2019). The small intestine and colon are complex organs composed of multiple muscle layers, an overlying submucosal layer, and a single, inner layer of epithelial cells, all innervated by the enteric nervous system and surveilled by the innate and adaptive mucosal immune systems. The epithelial cell layer of the small intestine and the colon interfaces with the external environment through luminal interactions with nutrients and the microbiota at the apical cell surface and the internal environment through interactions with immune cells, the enteric nervous system, and the blood circulation on the basolateral side (Figure 1). The small intestine and colonic epithelium are each composed of a variety of specialized cell types, including stem cells, one population of which is marked by Lgr5+ expression and dependent on Wnt signaling, that constantly regenerate the epithelium, goblet cells that produce protective mucus (Specian & Oliver, 1991), hormone‐secreting enteroendocrine cells (Dahly et al., 2003; Koehler et al., 2015; Koopmann et al., 2010), tuft cells that integrate signals from the enteric nervous and immune systems with the luminal environment (Gerbe et al., 2011; von Moltke, 2018), and absorptive enterocytes (small intestine) or colonocytes (colon) (Beumer & Clevers, 2021). In the small intestine, additional specialized cells include antimicrobial peptide‐producing Paneth cells (Bevins & Salzman, 2011; Bjerknes & Cheng, 1981), immune‐associated M cells (Owen & Jones, 1974), and pH‐sensitive BEST4 cells (Burclaff et al., 2022; Ito et al., 2013; Malonga et al., 2024). Each type of intestinal epithelial cell (IEC) plays a distinct role in contributing to barrier function and innate immunity. Lineage specification of each cell type and its role in health and disease are defined through complex signaling pathways and environmental determinants. Whether certain cell types are differentially influenced by EVs is largely an open question.

The GI tract is one of the few organ systems exposed to internally‐ and environmentally derived EVs. Internal EVs can emanate from any organ system in the body and are disseminated through the circulation. External or environmental EVs come from bacterial and nutrient sources (Figure 1).

This review will focus on EVs and their role in gastrointestinal health and disease. We will cover what is known about EV biology in shaping gut development and maturation, roles in homeostatic health, and examine what is known about nutritionally derived EVs and how they interact with cells of the GI tract. This review will also discuss EVs in intestinal inflammation, including necrotizing enterocolitis (NEC) in infants and inflammatory bowel diseases (IBD) Crohn's and colitis in pediatric or adult patients. We will discuss the beneficial and detrimental roles of bacterial‐derived EVs on the host gut and immune response and how the power of EVs is being harnessed for oral drug delivery. Throughout the review, we will point out key unanswered questions and areas where further research is needed.

## 2 INTERNAL EVs

Most studies to date examine how gut‐derived EVs (bacterial, nutrient, and immune) impact other organ systems. There are a few reports of the converse, where EVs secreted by distant organs or other tissues interact with or alter intestinal physiology. One recent report demonstrates that normal pancreas‐derived EVs are taken up by the small intestine and colon (Adem et al., 2024). How these EVs affect gut physiology remains to be defined. There is currently no evidence that EVs secreted by the liver or adipose tissue impact healthy intestinal function during homeostasis, but there is some evidence indicating that EVs found within the intestine can impact liver pathophysiology, including nonalcoholic fatty liver disease (Tong et al., 2023), as well as evidence indicating that EV‐mediated crosstalk between the intestine and adipose tissue contributes to tissue inflammation (Wei et al., 2020) and insulin sensitivity (Luo et al., 2021). Intestinal‐derived EV cargo is influenced by diet and metabolic state (Ferreira et al., 2022); however, if and how these EVs impact whole‐body or organ‐specific physiology is unknown. Much of what is known about internal EV—gut crosstalk involves communication with immune cell populations altering the inflammatory state of the intestine (Bauer et al., 2022). EVs secreted by dendritic cells, polymorphonuclear cells (PMNs), macrophages, and granulocytic myeloid‐derived suppressor cells (G‐MDSC) carry cargo that helps ameliorate or potentiate gut inflammation, as discussed in more detail below. Furthermore, IEC‐derived EVs can signal in an autocrine manner to other IECs. Work by Bakirtzi et al. shows that neuropeptide substance P stimulation of colonic epithelial cells stimulates miR21 cargo enrichment and EV secretion in vitro and in mice. These EVs can then act locally on other colonocytes to promote proliferation and migration (Bakirtzi et al., 2019). Whether these miR‐21‐enriched EVs are sufficient to induce proliferation in the absence of substance P was not tested. Substance P is secreted during inflammation, indicating that this may be a protective mechanism to respond to local tissue damage. Finally, there is accumulating evidence that the GI epithelium responds to mesenchymal stem cell‐derived‐EVs (MSC‐EVs), which aid in healing in response to damage, also discussed further below.

## 3 EXTERNAL EVS

The intestinal and colonic epithelium is exposed to myriad EVs from bacteria, plants, and other nutrient sources. These EVs target the epithelial cells themselves or the associated immune system, where evidence indicates they can attenuate inflammation. The most widely studied dietary‐derived EVs come from milk and a variety of plant species.

EVs are abundant in milk across many mammalian species, including bovine, porcine, murine, rat, and human. Milk EVs carry numerous proteins (Larssen et al., 2017; Vahkal et al., 2024; van Herwijnen et al., 2016; Wang et al., 2019; Yang et al., 2017), RNAs (Kahn et al., 2018; Kosaka et al., 2010; Kupsco et al., 2021; Liao et al., 2017; Vahkal et al., 2024; van Herwijnen et al., 2016; Zhou et al., 2012), and lipids (Miklavcic et al., 2024) to be taken up by epithelial cells within the GI tract (Sukreet et al., 2023; Tong et al., 2023; Yung et al., 2024) where they can influence gene expression or cellular function (He et al., 2021; Lu et al., 2024; Munir et al., 2024). Notably, an 80% reduction in milk EV production in a mouse model lacking the EV protein Tsg101 in mammary tissue led to diminished intestinal barrier function and reduced expression of barrier proteins, demonstrating a direct link between milk EVs and postnatal intestinal maturation in mice (Munir et al., 2024). These beneficial effects could in part be driven by miR‐146a‐5p, which was recently found in porcine and murine milk EVs and was enriched in response to a fiber‐rich diet. miR‐146a‐5p delivery led to Wnt‐mediated IEC proliferation because of miR‐146a‐5p‐driven NEDD4L degradation (Lu et al., 2024). Enhanced IEC proliferation can be beneficial and reparative in the setting of epithelial turnover during increased epithelial cell death. Milk EVs are most widely studied for their beneficial impacts on the immune (Zonneveld et al., 2021) and epithelial cells of the GI tract and their potential for oral therapeutic delivery, as detailed in subsequent sections.

Dietary EVs are also prevalent in plant foods. Grapefruit (Wang et al., 2014), red cabbage, (Kang et al., 2024) and ginger (Zhang et al., 2016)—derived EVs are taken up by intestinal macrophages where they limit inflammation in THP1 macrophages (Kang et al., 2024), mouse models of colitis, (Deng et al., 2017; Kang et al., 2024; Wang et al., 2014; Zhang et al., 2016) and colitis‐associated cancer (Zhang et al., 2016). EVs from broccoli can be absorbed by intestinal dendritic cells (Deng et al., 2017) where they stimulate adenosine monophosphate (AMP) activated protein kinase (AMPK) signaling and limit inflammation in a mouse model of colitis. The authors nicely demonstrated that AMPK is required for anti‐inflammatory protection, as dendritic cells from *AMPKa1* ^*−/−*^ mice failed to show a beneficial effect (Deng et al., 2017).

Ginger‐ (Mu et al., 2014), grape‐ (Ju et al., 2013), red cabbage‐ (Kang et al., 2024), and carrot (Mu et al., 2014)–derived EVs are taken up by IECs. Grape and carrot EVs influence Wnt signaling, with grape EVs selectively targeting Lgr5‐intestinal epithelial stem cells, where they promote proliferation. This elevated proliferation could be why treatment with grape EVs also reduced colitis symptoms in DSS‐treated mice (Ju et al., 2013). Red cabbage‐derived EVs promote intestinal barrier function through upregulation of occludin and zonula occludins‐1 (Kang et al., 2024).

Plant‐derived EVs also indirectly impact intestinal inflammation through interactions with the gut microbiome. For example, work from Teng et al. shows that ginger‐derived EVs interact with *Lactobacillus rhamnosus to* modulate the microbial community, promote aryl hydrocarbon receptor and IL‐22 signaling, and limit inflammation in a mouse model of colitis (Teng et al., 2018). Notably, ginger‐derived EVs also selectively promote the growth of *L. reuteri* and *L. murinus* (Teng et al., 2018). In vitro evidence indicates that watermelon‐derived EVs may promote fetal growth by enhancing placental development. The authors propose this mechanism involves alteration of the mother's intestinal metabolome through uptake of watermelon EVs, benefits which could then be transferred to the placenta through IEC secretions, tested here through conditioned media transfer (Timms et al., 2022). Further testing in in vivo systems is required for more definitive conclusions, but the data are provocative, indicating potential dietary EV‐mediated benefits on fetal health and development.

**FIGURE 2:**
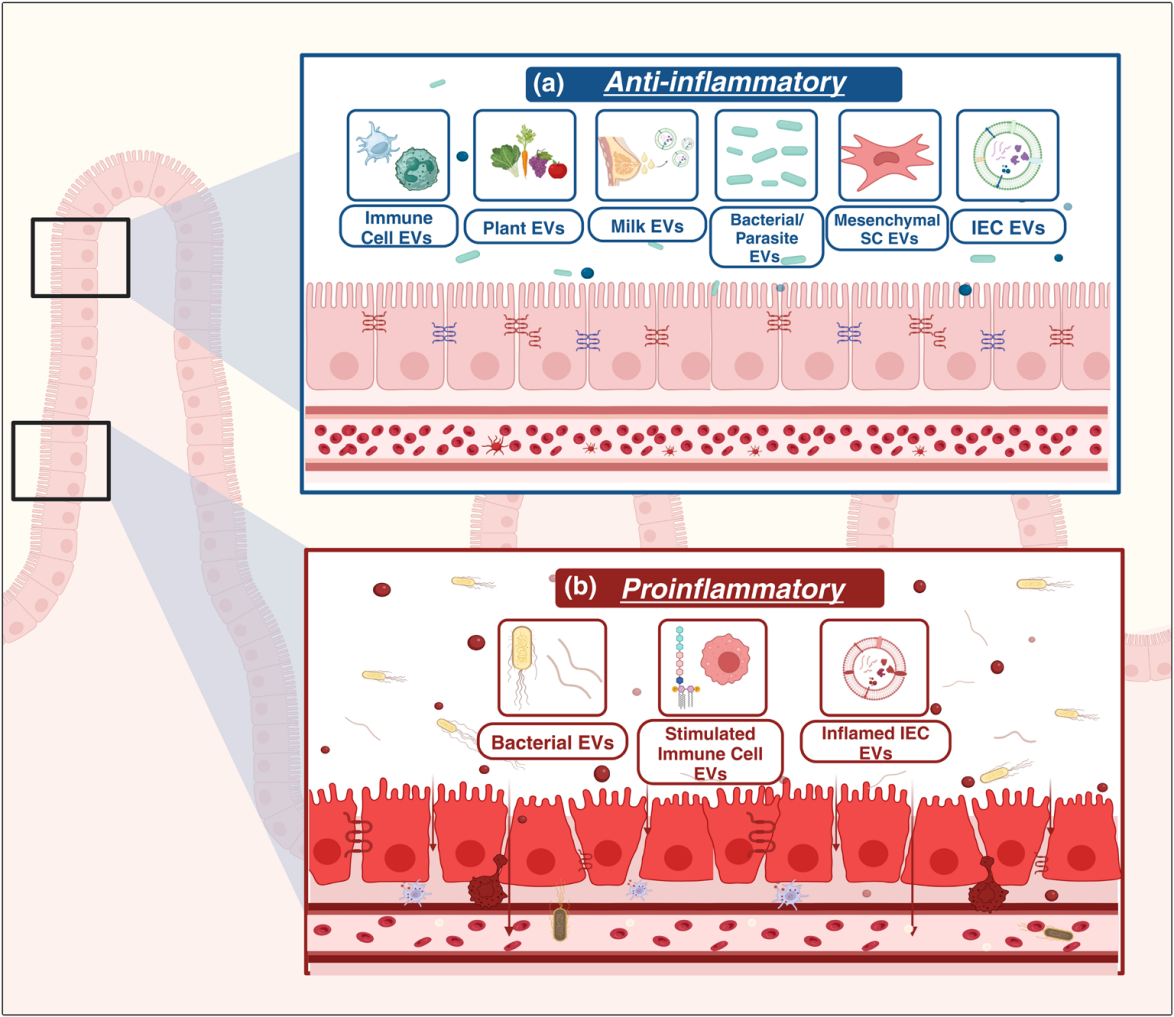
Extracellular vesicles (EVs) have pro‐ and anti‐inflammatory effects in the GI tract dependent upon the source and context. (a) Many EVs can be anti‐inflammatory and beneficial during intestinal inflammation. EVs from dendritic cells and neutrophils interact with IECs or pathogenic fungi, respectively, to resolve inflammation. Plant‐derived EVs have many anti‐inflammatory effects via effects on macrophages, dendritic cells, intestinal epithelial cells (IECs), or bacteria. Mesenchymal stem cell (SC) EVs can decrease inflammation via macrophages. Milk EVs interact with the immune and IECs to limit inflammation. The EVs of specific bacterial or parasites can also induce anti‐inflammatory effects in IECs. Finally, IEC‐derived EVs can act locally to attenuate inflammation. (b) On the contrary, EVs from these same sources in different contexts can exacerbate inflammation. EVs from pathogenic bacteria act on macrophages or IECs to potentiate inflammation. Lipopolysaccharide from gram negative bacteria can stimulate macrophages to produce pro‐inflammatory EVs that act on IECs. IECs themselves can promote their inflammation in an autocrine manner through IEC‐EV interactions. Collectively, these findings indicate that the cell type and context in which EV production and secretion occur have a sizable impact on EV cargo and target tissue response.

Collectively, these results indicate that dietary EVs can possess properties that promote uptake by specific IECs to promote intestinal turnover and reduce inflammatory disease (Ju et al., 2013) (Figure 2a). Which EV cargoes contribute to specific beneficial effects or cellular targeting is incompletely defined. Many of the plant‐derived EVs share high levels of phosphatidylethanolamine (Ju et al., 2013; Wang et al., 2014) and phosphatidic acid PEVuZE5vdGU (Ju et al., 2013; Zhang et al., 2016), which might contribute to uptake or targeting. Notably, plant‐derived vesicles offer a relatively cheap, low allergy risk, and plentiful source of vesicles for oral drug delivery (Corvigno et al., 2024). Cargo loading of specific drugs, such as methotrexate, demonstrates a use for dietary‐derived EVs in targeting specific GI‐associated cell types. The precise mechanisms governing reduced inflammation with EV treatment remain to be determined (Wang et al., 2014).

Importantly, there is debate about the bioavailability of nutritionally derived EVs and their ability to transfer their cargo to organs beyond the intestine. This debate is beyond the scope of this gut‐centric review, but we direct interested readers to a recent book chapter by Sanwlani et al. (Sanwlani et al., 2021).

## 4 EVs DURING (GUT) DEVELOPMENT

There is not much known about the role of EVs on embryonic intestinal development. Yet recent exciting research indicates that the maternal fecal microbiome may prime the infant gut for immune tolerance during early bacterial colonization via fecal‐derived microbial EVs. Kaisanlahti et al. demonstrated that maternal fecal and amnionic fluid EVs have a large degree of overlap in their 16S gene analyses, indicating shared bacterial origin. To determine if fecal EVs could traffic to a developing fetus, the authors isolated fecal EVs from pregnant humans and injected them via the tail vein into pregnant mice. The fluorescently labeled EVs were found in the developing fetus 24 h later, indicating that fecal EVs that enter the circulation can travel to the fetus and highlighting the potential for fecal EVs to interact with the developing gut during the swallowing of amnionic fluid. Fecal EVs could provide a non‐cellular/non‐replicative source of immunogenic material to safely prime the fetal immune system for immunotolerance (Kaisanlahti et al., 2023).

## 5 HOST—MICROBE INTERACTIONS VIA EVs

Host‐derived EVs play a crucial role in shaping the intestinal microbiota in both healthy and disease conditions. MiRNAs, primarily contained within EVs secreted by IECs, can enter bacteria, regulate their gene expression, and influence their growth by binding to nucleic DNA (Liu et al., 2016). Moreover, mice unable to release these miRNAs from IECs developed dysbiosis and were more susceptible to colitis, which was reversed by transplanting fecal miRNAs from wild‐type mice (Liu et al., 2016). In colorectal cancer, 76 miRNAs were differentially expressed in EVs from tumor tissues compared to normal tissues, correlating with changes in intestinal bacterial populations, including *Firmicutes*, *Bacteroidetes*, and *Proteobacteria* (Yuan et al., 2018), although a causative role of tumor‐released EVs in determining gut microbiota composition has not yet been demonstrated. Similarly, during fungal infections, EVs enriched with antimicrobial proteins are released from gut mucosal neutrophils, and these EVs bind to fungi, inhibit their growth, and cause damage in a host response to remove the pathogen (Shopova et al., 2020).

In addition to host‐derived EVs acting on the gut microbiome, the reciprocal also occurs with bacterial EVs (termed outer membrane vesicles, OMVs) activating host immune and IEC responses. Bacterial OMVs can exert their effects by delivering RNA into host epithelial cells, where it reaches both the cytoplasm and nucleus (Blenkiron et al., 2016), thereby providing exogenous gene transcripts that influence the global transcriptome of the cell. Additionally, bacterial OMVs interact with pathogen‐associated molecular pattern (PAMP) receptors of recipient cells, triggering inflammatory responses, including NF‐κB activation and the production of proinflammatory cytokines like tumor necrosis factor (Tnf), interfeuron gamma (IFN‐γ), IL‐6, and IL‐8 (Canas et al., 2018; Engevik et al., 2021; Pathirana & Kaparakis‐Liaskos, 2016) (Figure 2b). OMVs derived from pathogens carry virulence factors, toxins, and immunomodulatory effectors that enable bacteria to target host cells, disrupt host functions, and evade the immune system (Kaparakis‐Liaskos & Ferrero, 2015; Liu, Hsieh, et al., 2019; Zhang, Wang, et al., 2023). For example, OMVs released from *Fusobacterium nucleatum* were shown to significantly contribute to the loss of epithelial barrier integrity, increase oxidative stress in macrophage/Caco2 co‐culture conditions (to mimic intestinal environment), and aggravate dextran sodium sulfate (DSS)‐induced acute colitis (Beumer & Clevers, 2021). These effects are likely linked to IEC necroptosis, potentially driven by the activation of receptor‐interacting protein kinase 1 and receptor‐interacting protein kinase 3 (Liu et al., 2021). Additionally, *F. nucleatum* OMVs promote joint inflammation in rheumatoid arthritis (Hong et al., 2023).

Bacterial OMVs can also interact with the host and exhibit protective properties (Figure 2a). *Akkermansia muciniphila* OMVs promote intestinal homeostasis via regulation of the microbiota, mucosal immune system, and stimulating mucus secretion (Wang, Lin, et al., 2023). OMVs produced by *Bifidobacterium longum*, a beneficial bacterium, were demonstrated to package and export mucin‐a proteins which helped *B. longum* adhesion and survival in the mice gut lumen (Nishiyama et al., 2020). Mice injected with EVs from the roundworm, *Nippostrongylus brasiliensis* showed reduced colitis, likely due to miRNAs that suppress proinflammatory cytokines and promoted the production of anti‐inflammatory IL‐10 (Eichenberger et al., 2018). Overall, the host constantly communicates with the microbiota through EVs by regulating the immune response, intestinal barrier function, and bacterial colonization. Moreover, the contents within these EVs are not passively packaged because they vary depending on different conditions; instead, they are actively regulated by unknown mechanisms (Dixson et al., 2023; Kumar et al., 2024). Recent work using *Bacteroides thetaiotaomicron* indicates that the presence of different glycan substrates influences which glycan degrading enzymes are packaged into bacterial OMVs and which are maintained within the bacterial cell (Sartorio et al., 2023). This suggests that bacteria can cater to the environment to assist with substrate breakdown, making these nutrients available to the host.

For the sake of space, we chose to highlight the roles of OMVs from the pathogen *F*. *nucleatum* and commensals *B. theta* and *B. bifidum*. For more information about OMVs, we refer the reader to the following (Diez‐Sainz et al., 2022; Niu et al., 2023; Tian et al., 2023; Wu et al., 2024; Zhang et al., 2024; Zhao & Jones, 2023).

## 6 EVs IN INFLAMMATION

### 6.1 EVs in necrotizing enterocolitis (NEC)

NEC is a deadly multifactorial disease primarily affecting preterm infants. NEC is characterized by intestinal inflammation, injury, and necrosis (Rich & Dolgin, 2017). NEC has an estimated mortality rate of around 20%–30% but increases to 50% in cases that require surgical intervention (Jones & Hall, 2020). Patients who survive NEC are at an increased risk for long‐term complications, including short bowel syndrome and neurodevelopmental defects (Bazacliu & Neu, 2019; Rees et al., 2007).

Current models suggest NEC arises from some combination of risk factors including intestinal injury such as hypoperfusion, ischemia, and perforation; intestine developmental immaturity; and microbial dysbiosis (Kelleher et al., 2021; Pammi et al., 2017). For a more complete review of conditions associated with NEC, please refer to “A Critical Analysis of Risk Factors for NEC” (Rose & Patel, 2018).

While many factors are associated with NEC, there is no clear consensus on the disease pathophysiology or reliable predictors for which infants will develop NEC. Prophylactic treatment for NEC includes protective probiotics (Chi et al., 2021; Morgan et al., 2020; Sharif et al., 2023) and stimulation of gut maturation through various methods, including human milk feeding (Altobelli et al., 2020; Li et al., 2022; Quigley & McGuire, 2014; Strobel et al., 2022).

Recent research suggests that EVs from several sources can be used to protect against the occurrence of NEC or lessen the impact in tissue culture and animal models (Figure 2a). This research can broadly be categorized by the source of EVs used. First are EVs from human milk (HM) and milk from animal models. EVs are one of the many signaling components of HM (Andres et al., 2023) and since HMEVs have been shown to survive digestion and are absorbed by intestinal cells (Kahn et al., 2018; Larssen et al., 2017; Liao et al., 2017; Yung et al., 2024), they are proposed to be a facet of protective mechanisms (Hock et al., 2017). Secondly, there is evidence that stem cell (SC)‐derived EVs are beneficial. In 2011, Tayman et al. were the first to show that SC implantation could reduce disease severity in a rat model of NEC (Tayman et al., 2011). However, Rager et al. later showed that SC‐EVs could offer similar protection, suggesting the original effect was mediated by SC‐EVs (Rager et al., 2016). Data indicate that EVs from many different SC types can protect against NEC, including amniotic fluid SCs, mesenchymal SCs, bone marrow SCs, placental EVs, and neonatal enteric neural SCs (McCulloh et al., 2018; O'Connell et al., 2021) (Figure 2a). HMEVs and SC‐EVs are linked by their role in development, growth, immune protection, and regeneration, all of which promote beneficial outcomes in NEC.

There are many different mechanisms proposed for the positive impact of HMEVs and SC‐EVs in NEC. Given the complexity of EV cargos, it is likely a combination of many pathways. EVs can increase IEC proliferation and viability (Chen et al., 2016; Dong et al., 2020; Hock et al., 2017; Li et al., 2020; Miyake et al., 2020; O'Connell et al., 2021; Pisano et al., 2020). These effects may be mediated in part by EV activation of the Wnt pathway, as EV‐mediated protection against H_2_O_2_‐induced IEC damage and experimental NEC was Wnt‐dependent (Dong et al., 2020; Li et al., 2019, 2020).

Data indicate that milk and SC‐EVs can also modulate immune responses in experimental NEC. Application of EVs can decrease markers of inflammation, including IL‐1b, 6, 7a, 18, CD40, TNF‐a, and increase expression of anti‐inflammatory IL‐10 in animal models of NEC (Dong et al., 2020; Gomez‐Ferrer et al., 2023; Li et al., 2020; Miyake et al., 2020; Tong et al., 2023). Some evidence suggests that this inflammatory regulation of NEC is COX2‐dependent (Gomez‐Ferrer et al., 2023; Zani et al., 2014). NEC is also characterized by diminished gut barrier integrity, which can lead to sepsis (Duess et al., 2023). Studies indicate that milk EVs increase goblet cell mucus production and increase expression of epithelial barrier proteins like occludin and Zonula Occludens‐1 (ZO‐1) in animal models of NEC (Gomez‐Ferrer et al., 2023; Miyake et al., 2020; Tong et al., 2023) to protect or promote barrier function. Moreover, milk EVs also promote intestinal barrier function in a mouse model of barrier breakdown caused by malnutrition (Maghraby et al., 2021).

### 6.2 EVs in inflammatory bowel disease (IBD)

IBD, including Crohn's disease and ulcerative colitis, is a chronic relapsing inflammatory disease of the GI tract. Approximately 5 million people are afflicted with IBD worldwide, with continued increasing incidence and prevalence rates depending on geographical location (Wang, Li, et al., 2023). The etiology of IBD is thought to involve the complex interaction of multiple factors, including environmental and microbial triggers, genetic susceptibility, and the immune response. IBD commonly presents with abdominal pain, diarrhea, and rectal bleeding (Yu & Rodriguez, 2017). Pathological changes within the intestine include the manifestation of inflammation, epithelial barrier dysfunction, and microbiota dysbiosis (Hu et al., 2023; Saez et al., 2023). Current treatment strategies for IBD target the immune response/signaling, but a “therapeutic ceiling” consisting of lacking or loss of therapeutic response is demonstrated in IBD patients (Raine & Danese, 2022).

The role of EVs in IBD emerged largely in recent years. Studies show that EV concentration increases in the serum or peripheral blood of IBD patients (Caparros et al., 2024; Gong et al., 2022; Leonetti et al., 2013; Leoni et al., 2015). Furthermore, the cargo profile of IBD‐derived EVs was altered, including differential expression of miRNAs (Caparros et al., 2024; Gong et al., 2022), long noncoding RNAs (Heydari et al., 2024), double‐stranded DNA including both mitochondrial and nuclear genomic DNA that activated inflammatory signaling (Zhao et al., 2021), and mRNA and protein levels of cytokines IL‐6, IL‐8, IL‐10, and TNFα (Mitsuhashi et al., 2016). Like NEC, data indicate that IBD‐derived EVs can modulate immune responses (Figure 2). For instance, IBD‐derived EVs or EVs from mouse models of colitis induced immune cell activation of macrophages and neutrophils and immune‐mediated disease processes compared to EVs derived from healthy cells (Hu et al., 2021; Ma et al., 2023; Zhang, Zheng, et al., 2023).

Preclinical and in vitro models suggest varying effects of EVs on IBD depending on their cellular source (Chen et al., 2023) (Figure 2). EVs derived from dendritic cells treated with IL‐10 decreased the severity of experimental colitis in mice (Yang et al., 2010) and improved epithelial barrier function dependent on EV miR‐146b (Nata et al., 2013). EVs derived from macrophages exposed to lipopolysaccharide induced intestinal mucosal barrier dysfunction and worsened experimental colitis in mice (Chang et al., 2023). Commensal bacterial‐ or protozoan‐derived EVs exhibited therapeutic properties against colitis in mice, suggesting that commensal gut microbiota EVs may play a role in regulating intestinal inflammation (Hao et al., 2021; Kang et al., 2013; Kim et al., 2022). Additional therapeutic potential of EVs was shown by those derived from mesenchymal SC to reduce colitis in preclinical models and inflammatory cytokines in vitro (Heidari et al., 2021; Katifelis et al., 2022; Liu, Liang, et al., 2019; Zhang et al., 2022). EVs derived from IECs were isolated and studied from the DSS model of colitis (Jiang et al., 2016; Leoni et al., 2015) or cancer‐derived cell lines such as HT29, T84, or YAMC (Mallegol et al., 2007; van Niel et al., 2001). These IEC EVs exhibited an ability to interact with dendritic cells (Mallegol et al., 2007), promote epithelial wound healing (Leoni et al., 2015), promote immune balance between T effector and T regulatory cells via TGFβ1 (Jiang et al., 2016), and drive colitis (Bulek et al., 2020). Interestingly, EVs secreted from IECs of the small intestine and colon express A33 (Jiang et al., 2016), a cell surface glycoprotein specifically expressed in the intestinal epithelium (Williams et al., 2015). In contrast, EVs from sera, heart, liver, spleen, lung, and kidney are absent of A33 expression (Jiang et al., 2016). A33^+^ EVs were shown to traffic to GI organs but not other organs or blood, suggesting that IEC‐derived EVs maintain residence within the GI tract to perhaps elicit local responses (Jiang et al., 2016). The mechanism of this GI tract homing of IEC‐derived EVs remains to be defined.

Multiple studies indicate that during inflammation, macrophages are responsive to EVs, resulting in their polarization and activation. For example, EVs from IBD patients increase macrophage migration (Mitsuhashi et al., 2016) and polarization to pro‐inflammatory phenotypes (Hu et al., 2021; Ma et al., 2023). The protective effect of EVs derived from mesenchymal SC to reduce mouse colitis was dependent on the polarization of macrophages to an M2 phenotype, which acts to dampen inflammation and increase T regulatory cells (Cao et al., 2019; Heidari et al., 2021; Liu, Liang, et al., 2019). Serum exosomes from mice during DSS‐induced colitis activate macrophages to produce pro‐inflammatory TNFα (Wong et al., 2016). Similarly, bacterial infection with adherent‐invasive E. coli LF82, which is associated with Crohn's disease, induced the release of EVs from cultured IECs and macrophages, further inducing a pro‐inflammatory response in recipient macrophages and increasing bacterial replication (Carriere et al., 2016) (Figure 2b). These outcomes could result in both pro‐ and anti‐inflammatory responses in the tissue. Future studies are needed to better understand macrophage responses to EVs as being beneficial for intestinal homeostasis or exacerbating intestinal inflammation.

## 7 EVs FOR ORAL DELIVERY OF THERAPEUTICS

EVs possess unique properties that make them promising candidates for therapeutic applications. First, EVs are potentially less immunogenic than their parent cells, as they have lower levels of transmembrane proteins and are enriched with phosphatidylserine, which may help evade immune detection (Llorente et al., 2013; Ong & Wu, 2015). Second, EVs can enter nearby or distant recipient cells, facilitating the transfer of cargo and thereby triggering cellular responses (Andaloussi et al., 2013). Additionally, EVs can preferentially bind to specific cell types due to their distinct composition of proteins, lipids, glycans, and negatively charged phosphatidylserine, providing potential targeting of EVs to specific recipient cells (Berenguer et al., 2018; Hoshino et al., 2015; Matsumoto et al., 2017).

Given these properties, EVs from various sources, such as plants, milk, and bacteria have been shown to resist harsh conditions, including acidic and alkaline pH, allowing them to remain intact and be absorbed in the intestine (Benmoussa et al., 2016; Kahn et al., 2018; Liao et al., 2017; Shandilya et al., 2017; Tong et al., 2023; Yung et al., 2024). As a result, these EVs have been orally administered in studies to assess their therapeutic potential. In mouse models, plant‐derived EVs have shown immunoregulatory effects, preventing colitis by targeting intestinal macrophages and dendritic cells (Deng et al., 2017; Umezu et al., 2021; Wang et al., 2014). Similarly, orally administered milk‐derived EVs have been found to increase mucin secretion, modulate the gut microbiota (Benmoussa et al., 2019), protect intestinal barrier integrity (Tong et al., 2023), and reduce the severity of enterocolitis and colitis (Benmoussa et al., 2019; Reif et al., 2020). Bacterial‐derived EVs have also demonstrated the ability to regulate gut barrier function (Chelakkot, Choi, et al., 2018). This combination of low immunogenicity, targeted delivery, and therapeutic potential positions EVs as a novel approach to treating various GI disorders through vascular, or perhaps more targeted to the GI tract, via oral delivery. However, before introducing orally delivered EV‐based therapeutics into clinical practice, it is crucial to consider potential side effects. Orally delivered EVs have been detected in various organs, including the intestine, liver, spleen, kidneys, lungs, heart, and brain, potentially through circulation after absorption in the intestine. This biodistribution and accumulation in nontarget organs, along with challenges in shelf‐life and storage of EVs, could limit the therapeutic applications of oral EV delivery (Manca et al., 2018; Munir et al., 2023). Future studies are necessary to identify EV receptors that elicit homing to the GI tract, and more specifically, to individual cell types within the intestine to target EVs to desired intestinal recipient cells, thereby reducing systemic off‐target effects and enhancing therapeutic EV effectiveness.

## 8 SUMMARY AND OPEN QUESTIONS

EVs have broad implications on gut health and pathophysiology whether they are derived from internal or external sources. The EVs secreted by the intestine or from the microbial inhabitants also have far‐reaching implications on organismal health. There are several open questions in the field whose answers will advance the use of EVs in disease diagnosis and treatment. First, we need to develop a better understanding of cell‐specific EV targeting within the intestine and to distant organs outside the GI tract, including differential responses of EV signaling with the immune system and contributions to pro‐ versus anti‐inflammatory effects. Second, the field would benefit from a more detailed understanding of specific cargoes that induce physiological or pathological effects. Collectively, this will provide a more direct mechanistic understanding of many loosely defined EV‐associated outcomes. Third, it is important to examine roles for EV signaling during homeostasis and development, as understanding basic physiological functions may provide insight into pathological roles. Finally, when it comes to the use of EVs as therapeutics, we need to understand appropriate dosing, cell targeting, and off‐target side effects. Addressing these questions will pave the way to further use of EVs in personalized medicine and diagnostics.

## FUNDING INFORMATION

SFA is supported by the National Institutes of Health (NIH) grant K01DK129401, R01HD109193, and grants from the Gerber Foundation and the Knight Cancer Institute. ALT is supported by Veteran's Affairs BLRD MERIT IBX005288, Crohn's & Colitis Foundation SRA 900820, The Helmsley Charitable Trust 2505‐08294, and the GI & Liver Innate Immune Program (GALIIP) at the University of Colorado Anschutz. None of the funding sources had any role in the development of this manuscript.

## CONFLICT OF INTEREST STATEMENT

Dr. Theiss is an Associate Editor at Physiological Reports and was blinded from reviewing or making decisions for the manuscript. Another Editor oversaw the manuscript process for this article.

## ETHICS STATEMENT

This review article does not contain any original data and did not involve the use of human participants or animals; thus, no ethics approval was required.
